# Supplementary material for: Real-time decoding of question-and-answer speech dialogue using human cortical activity
Source: Nat Commun. 2019 Jul 30;10:3096. doi: 10.1038/s41467-019-10994-4 (PMC6667454; doi:10.1038/s41467-019-10994-4)
Supplement: Supplementary file 1 — Supplementary Information [file 41467_2019_10994_MOESM1_ESM.pdf]

Supplementary Information

Real-time decoding of question-and-answer speech dialogue using human cortical activity

Moses et al.

Supplementary figures

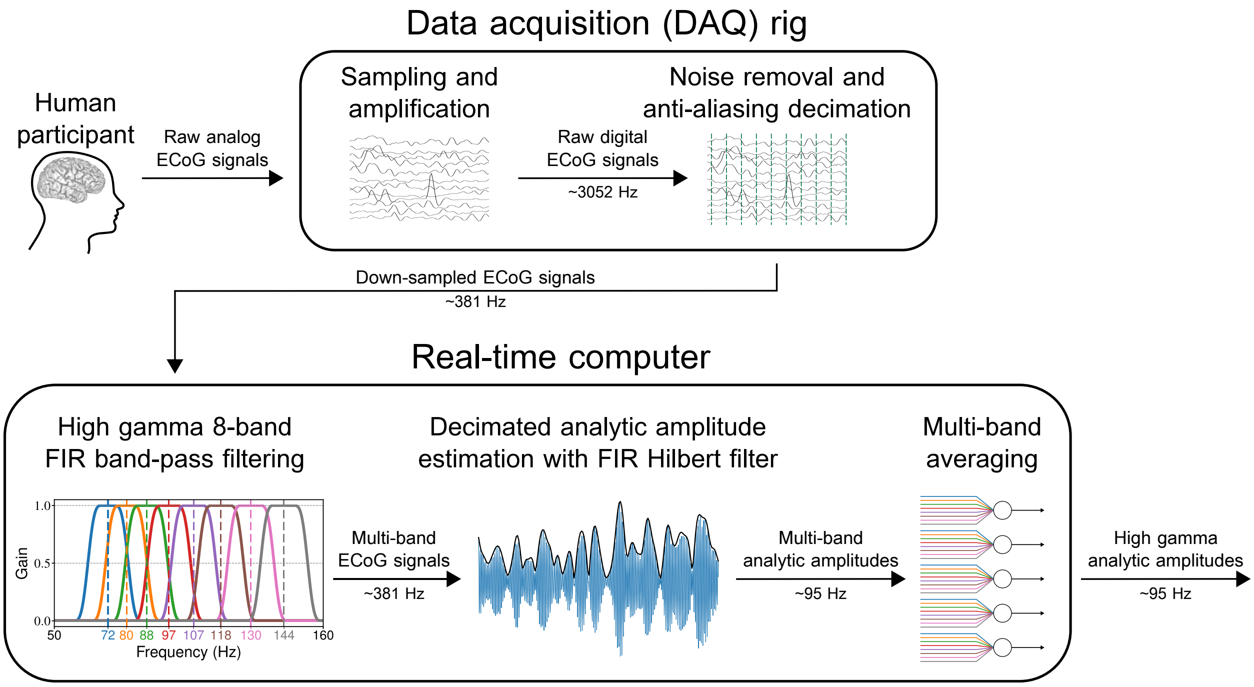

Supplementary Figure 1. Real-time neural signal preprocessing with the rtNSR system[1]. In the DAQ rig, ECoG signals are sampled from the participant's brain at ∼3052 Hz, quantized, notch filtered at 60, 120, and 180 Hz, and decimated (with anti-aliasing) to ∼381 Hz. The resulting signals are streamed into the real-time computer and, within rtNSR, band-passed using eight FIR filters with center frequencies in the high gamma band (filter responses shown in the bottom-left plot). The analytic amplitude is then estimated for each of the eight band-passed signals for each channel at ∼95 Hz using an FIR filter designed to approximate the Hilbert transform. The analytic amplitudes for the eight bands associated with each channel are averaged to yield a high gamma analytic amplitude signal for each channel.

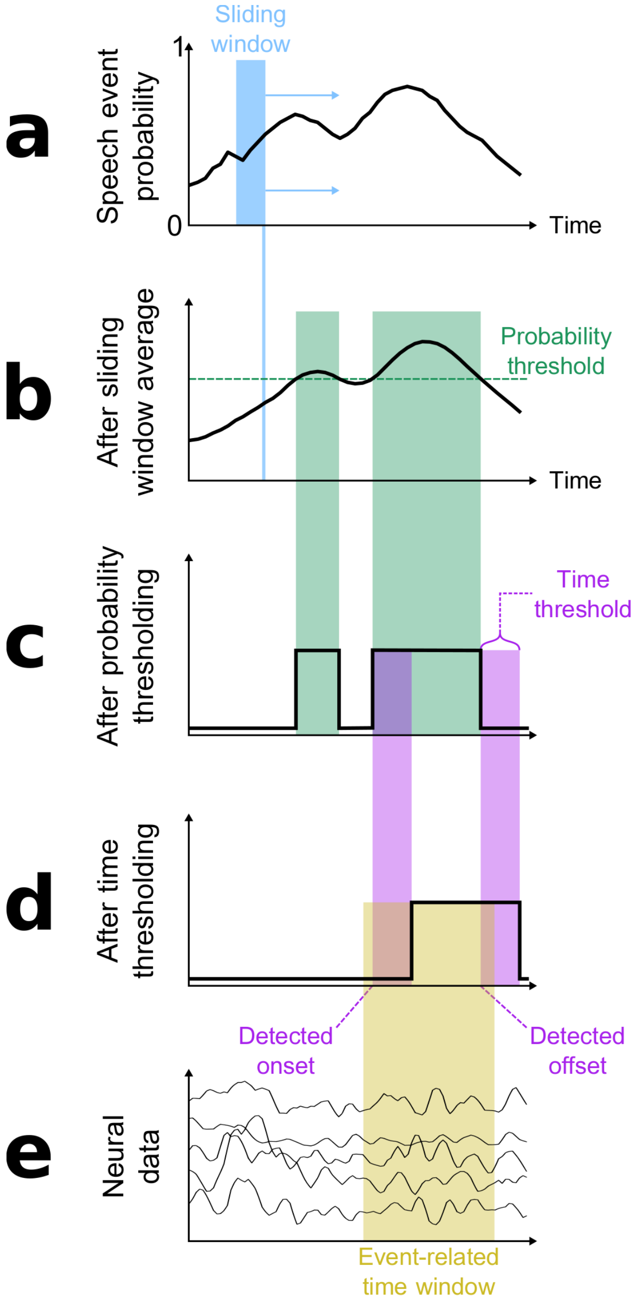

Supplementary Figure 2. Speech event detection during real-time decoding. (a) Speech event probabilities are computed by the detection model for each time point. The plotted curve depicts example event probabilities for one of the utterance types (for either question or answer events). (b) Speech event probabilities are smoothed using a sliding window average. (c) These smoothed probabilities are thresholded to be either 1 or 0. (d) These binary values are then thresholded in time. Sometimes referred to as debouncing, this step prevents false switches between binary states due to noise and the particular threshold chosen. A transition from 0 to 1 in the time-thresholded values signifies a speech onset, and a transition from 1 to 0 signifies a speech offset. (e) The neural data are segmented by the detected speech onset and offset, including some padded time points before and after the detected window (controlled by hyperparameters), and passed to the appropriate utterance classification model.

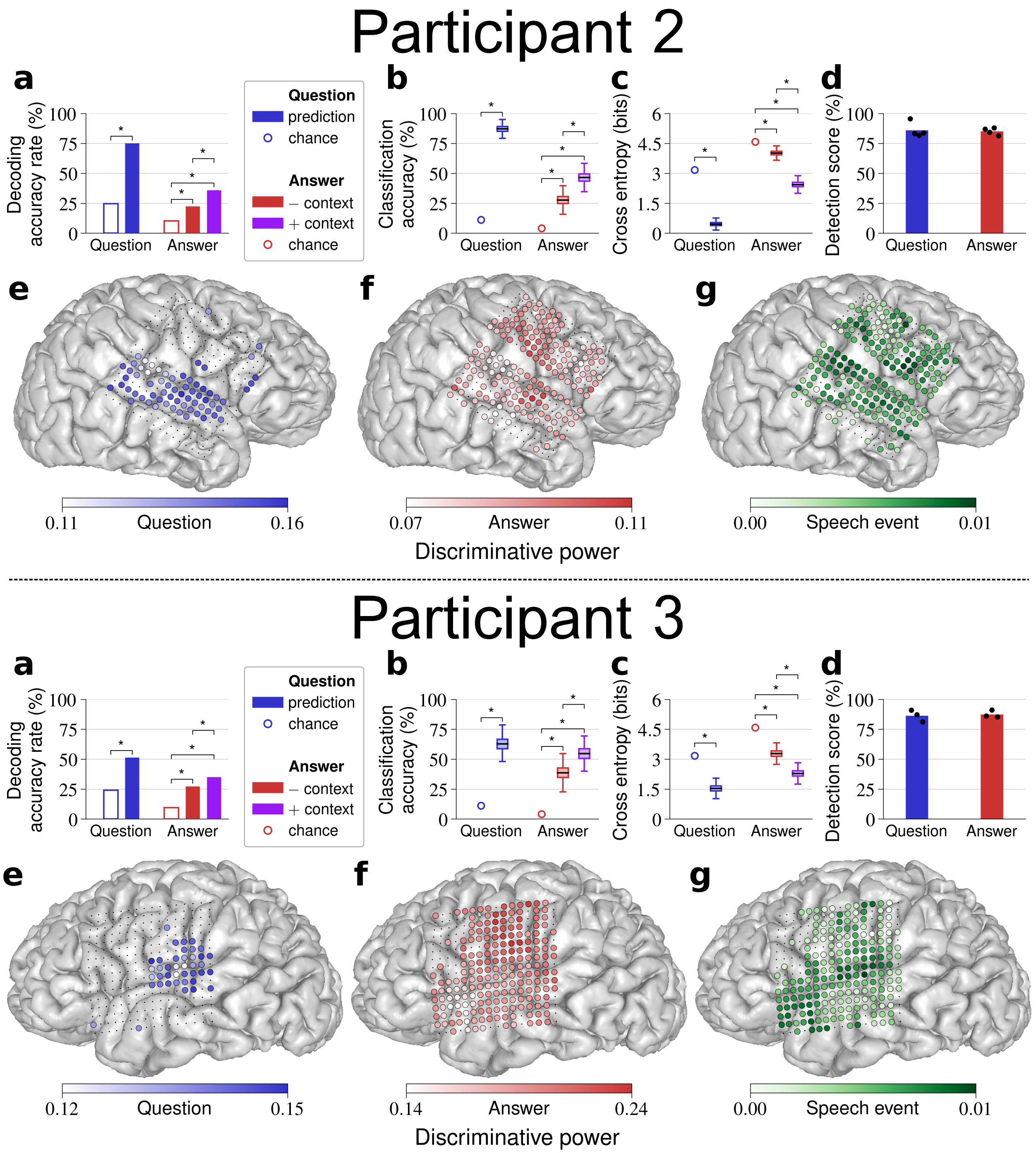

Supplementary Figure 3. Speech decoding and classification results for participants 2 and 3 (participant 1 shown in Fig. 2). (a) Decoding accuracy rate, which measures the full performance of the system, is significantly above chance for questions and answers (without and with context; * all $p < 0.05$, 4-way Holm-Bonferroni correction). Answer decoding accuracy rate is significantly higher with context compared to without context. (b) Classification accuracy (the percent of correctly classified speech events, using true event times) mirrors decoding accuracy rate. (c) Cross entropy for utterance classification demonstrates similar patterns of better-than-chance performance and improvement with context (lower values indicate better performance). In b–c, values were computed by bootstrapping across trials. Each boxplot depicts a line marking the median value, box heights representing the interquartile range, and whiskers extending beyond the box edges by 1.5 times the interquartile range. (d) Event detection scores demonstrate near-ceiling performance of the speech detection model for both questions and answers. Black dots depict detection scores on individual test blocks. (e–g) MRI brain reconstructions with electrode locations and discriminative power for each electrode used by e question, f answer, and g speech event discriminative models. Electrodes that were not relevant are depicted as small black dots.

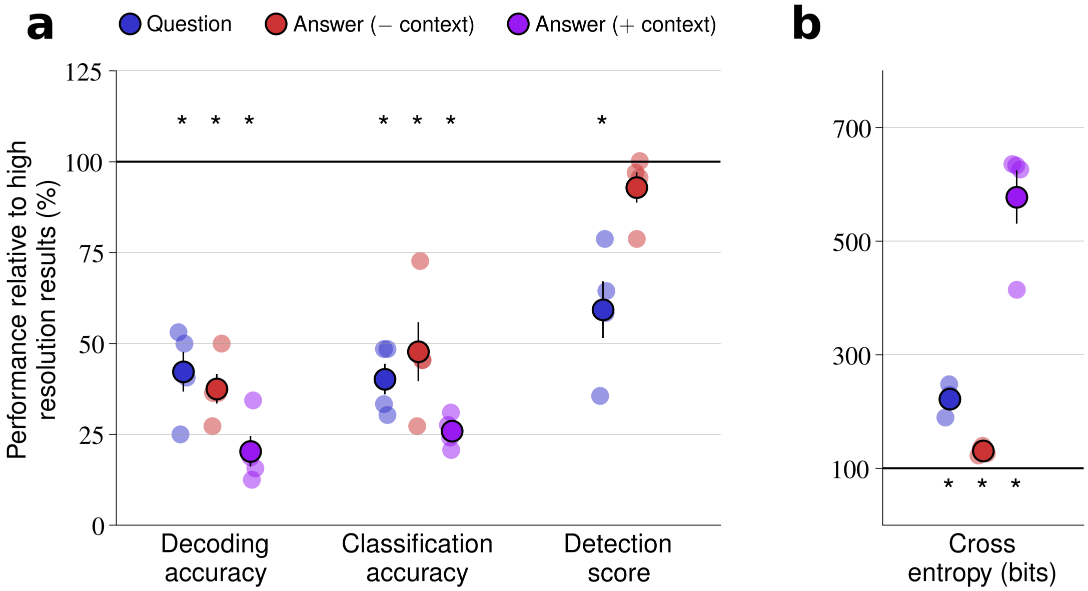

Supplementary Figure 4. Performance evaluation using simulated low-resolution spatial coverage with participant 1. All values are presented as percents of the corresponding high-resolution result value. Each dark dot (with black outline) depicts the performance (mean ± s.e.m.) across the four low-resolution simulation results (shown as light dots with no outline). Except for the answer detection score, performance is significantly worse with the low-resolution signals than with the high-resolution signals (* $p < 0.05$, one-tailed one-sample t-test).

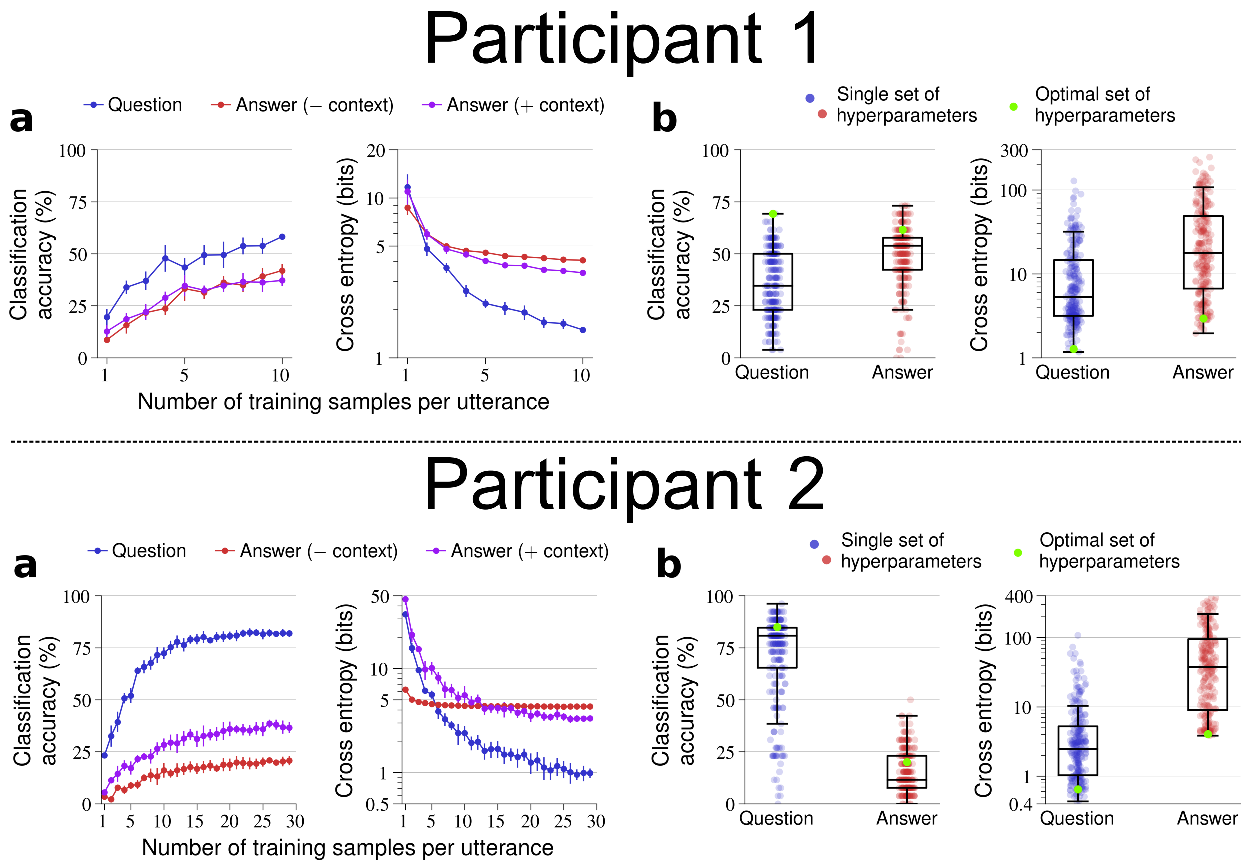

Supplementary Figure 5. Effects of amount of training data and hyperparameter optimization on speech classification for participants 1 and 2 (participant 3 shown in Fig. 3). (a) Classification accuracy and cross entropy as a function of the amount of training data (mean ± s.e.m.). (b) Variability in classification performance across hyperparameter optimization epochs for one test block with each participant. Each boxplot depicts a line marking the median value, box heights representing the interquartile range, and whiskers extending beyond the box edges by 1.5 times the interquartile range. Each blue and red dot shows the performance on the test block using a single set of hyperparameters chosen for one epoch during optimization on a separate validation set. Each green dot marks the performance on the test block using the hyperparameters that minimized cross entropy on the validation set (the hyperparameter values used in the main results).

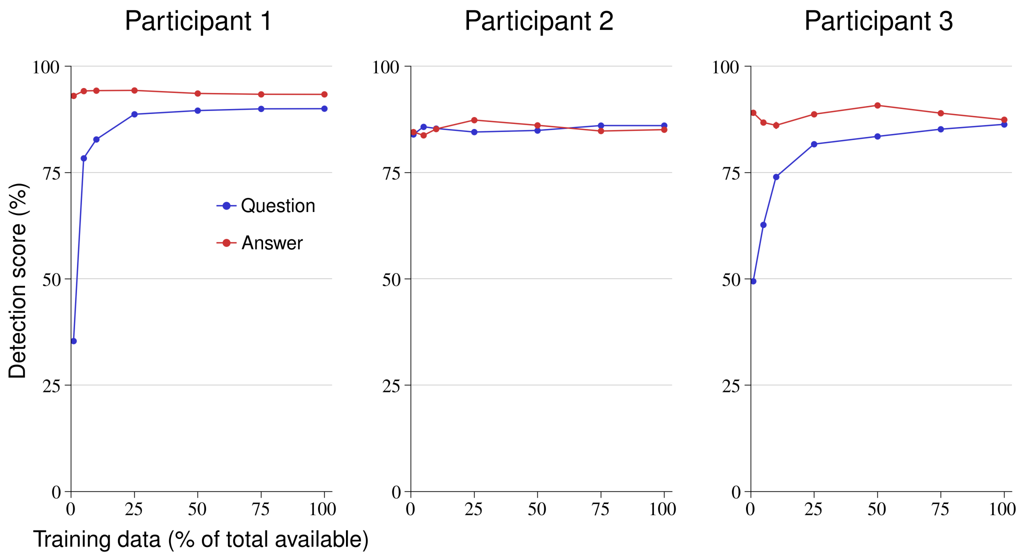

Supplementary Figure 6. Effects of amount of training data on speech detection for each participant. Each plot shows question and answer detection scores (mean ± s.e.m.) after fitting the speech detection models with various percents of the available speech and silence data points (between 1% and 100%). The percents in these plots are relative to the total amounts of available training data for each participant (shown in Supplementary Table 1). The error bars in this plot were typically too small to be seen alongside the circular markers.

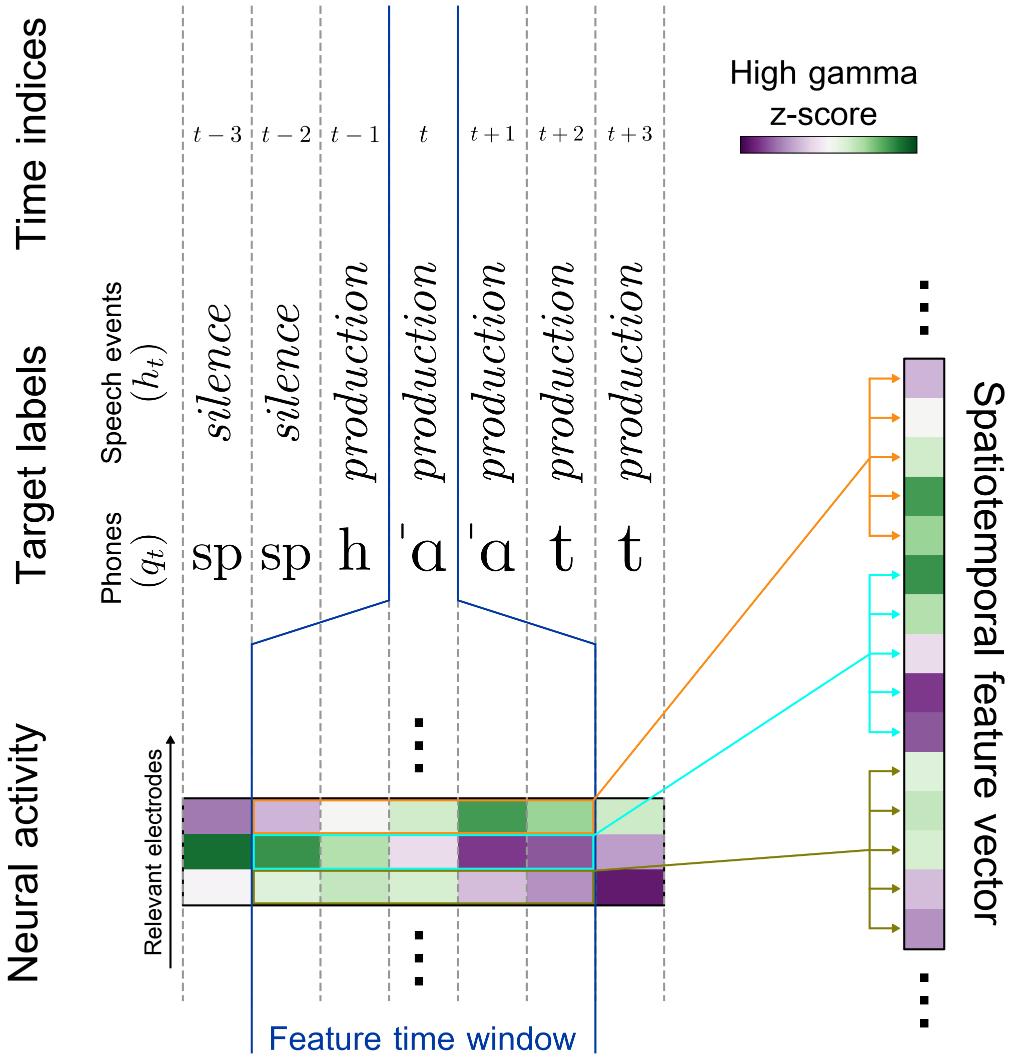

Supplementary Figure 7. Spatiotemporal neural feature vectors and associated target labels during training of the speech detection and utterance classification models. In this example, a participant produces the answer utterance "Hot" (with phonetic transcription /h ˈɑ t/). Speech onset occurs at time index $t-1$. The phone labels q_t at each time point t are obtained from phonetic transcriptions. The speech event labels h_t, which are either silence, perception, or production at every time point, are determined from these phonetic transcriptions. The feature vector at time t contains the high gamma z-score value at every relevant electrode for every time point within some feature time window relative to t. The feature vector and target label for each time index are used to train the speech event probability and phone likelihood models. During testing, the neural feature vectors y_t are constructed in a similar fashion and used within the speech detection model to compute the speech event probabilities $p\left(h_t|y_t\right)$ and within the utterance classification models to compute the phone likelihoods $p\left(y_t|q_t\right)$.

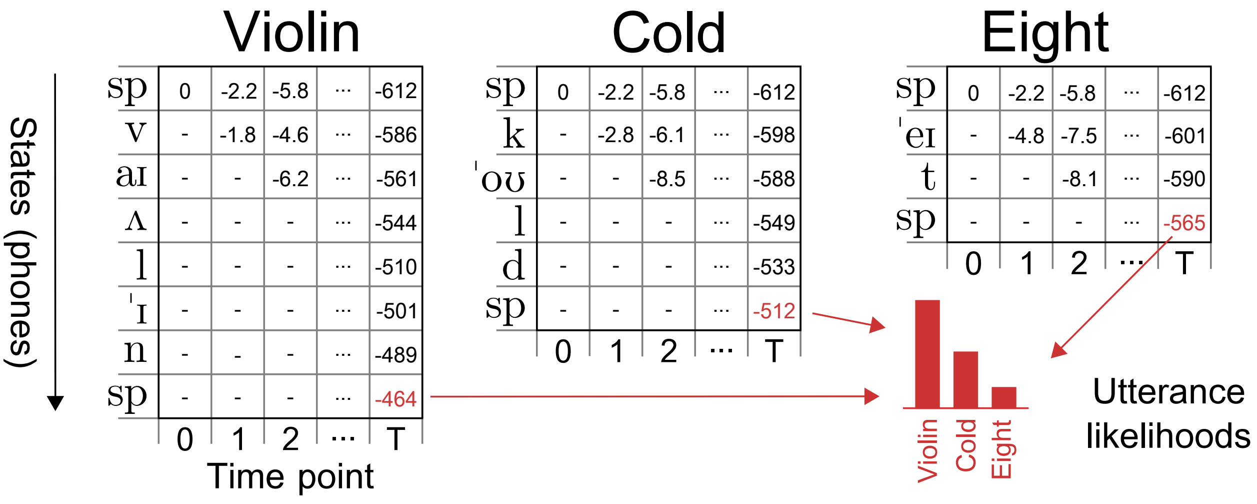

Supplementary Figure 8. Schematic example of Viterbi decoding in the utterance classification models. In this example, a classification model computes the likelihoods of the utterances "Violin" (/v aɪ ʌ l ˈɪ n/), "Cold" (/k ˈoʊ l d/), and "Eight" (/ˈeɪ t/). Each utterance is represented as an HMM with phones (obtained from the phonetic transcriptions) as hidden states and spatiotemporal neural feature vectors as observations. Each HMM is forced to have /sp/ as the first and last states. The transition matrix of each HMM is defined such that a phone state can only transition to itself or, if it is not the last phone, the next phone in the sequence. Given feature vectors for time indices $t \in \{0, 1, \ldots, T\}$, Viterbi decoding is performed on each HMM, updating the values in the Viterbi trellis for each HMM (shown here as tables of log likelihoods) at each time index. The log likelihood of the most likely Viterbi path at the final state of each HMM (the value for the final /sp/ state at time T) is used as the log likelihood of that utterance. The classifier then smooths and normalizes these log likelihood values to obtain a final estimate for the utterance likelihoods.

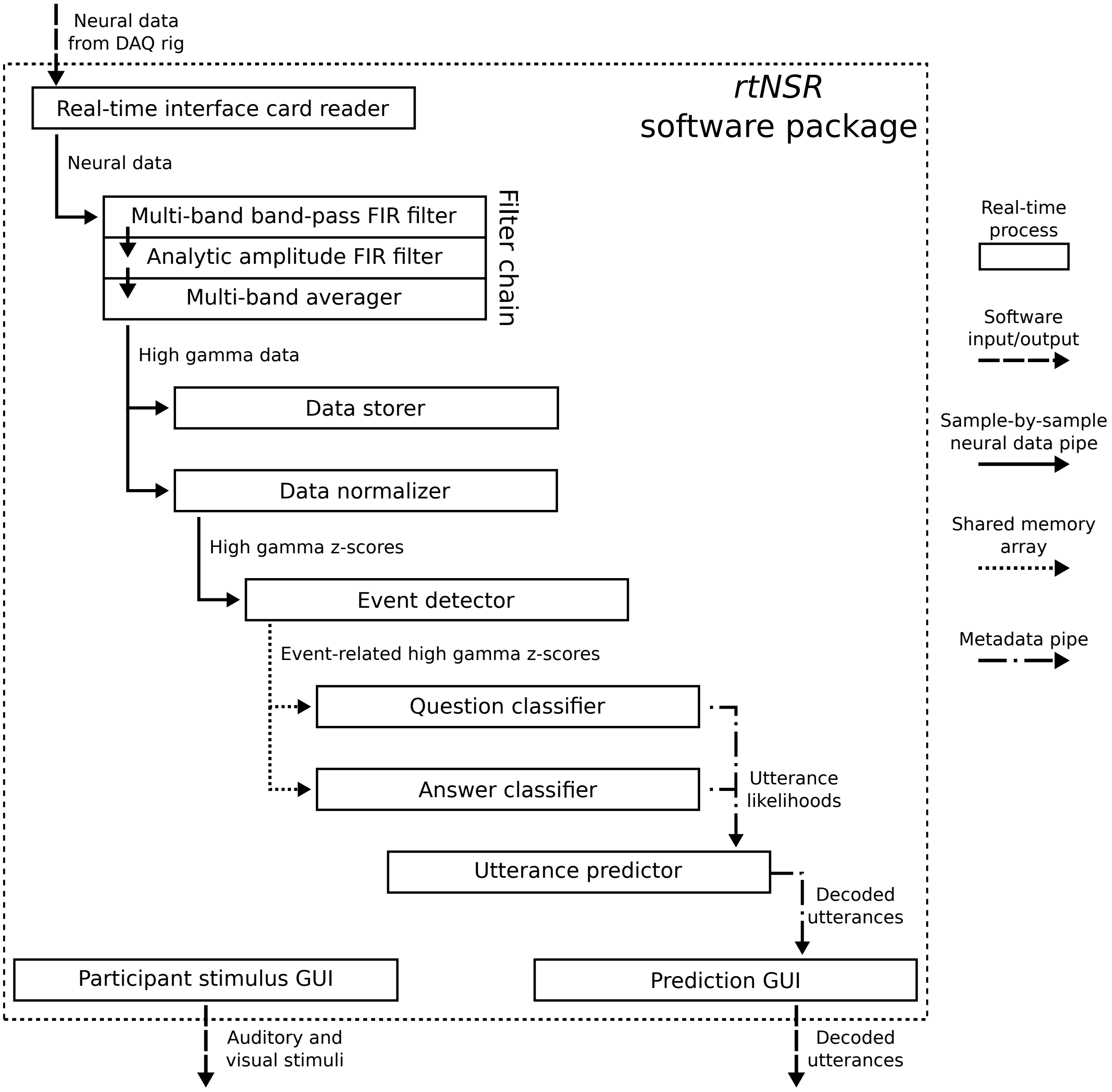

Supplementary Figure 9. Schematic depiction of the rtNSR system used during real-time decoding. The solid rectangles represent real-time process classes and arrows represent the passing of information between the processes. The *Real-time interface card reader* process reads neural data acquired from the DAQ rig and streamed through the real-time interface card (PO8e, Tucker-Davis Technologies). The neural data are processed in a filter chain comprising three processes: the *Multi-band band-pass FIR filter* process that band-passes the signals for each channel in eight different sub-bands in the high gamma band range (between 70–150 Hz), the *Analytic amplitude FIR filter* process that extracts the analytic amplitude for each band and each channel, and the *Multi-band averager* process that averages the analytic amplitude values across the bands for each channel to obtain the desired measure of that channel's high gamma activity. These high gamma signals are written to disk in the *Data storer* process (along with metadata from other processes, not depicted here) and normalized and clipped in the *Data normalizer* process. The normalized neural data are piped to the *Event detector* process, which analyzes the data at each time point to predict the onsets and offsets of speech events. When an event is detected, the high gamma z-scores are stored in a shared memory array that can be accessed by either the *Question classifier* or *Answer classifier* process to predict the utterance likelihoods associated with that event. The *Utterance predictor* process uses these likelihoods to update the answer priors and predict which question was heard or which answer was said by the participant. The *Prediction GUI* process displays the decoded utterances on a screen. Throughout the task, the *Participant stimulus GUI* process presents the auditory and visual stimuli to the participant.

Supplementary tables

Supplementary Table 1. Amount of training and testing data collected with each participant.

Participant	Data type	Total data	Question (perception) data[1]	Answer (production) data[1]
1	Training	3 task blocks; 941 seconds	90 trials; 189 seconds	240 trials; 134 seconds
	Testing	2 task blocks; 421 seconds	52 trials; 104 seconds	52 trials; 26 seconds
2	Training	9 task blocks; 2776 seconds	270 trials; 565 seconds	720 trials; 525 seconds
	Testing	4 task blocks; 1146 seconds	104 trials; 208 seconds	104 trials; 62 seconds
3	Training	6 task blocks; 1898 seconds	180 trials; 376 seconds	480 trials; 280 seconds
	Testing	3 task blocks; 875 seconds	78 trials; 156 seconds	78 trials; 40 seconds

[1] Each trial corresponds to one utterance, and the durations given here only consider time points that occurred during speech (silence time points are excluded).

Supplementary Table 2. Significance testing statistics for question and answer decoding and classification performance.

Metric	Test	Participant	Prediction type	p-value	Number of samples[1]
Decoding accuracy rate	One-tailed bootstrap test, performance vs. chance	1	Question	$< 1 \times 10^{-50}$	54
			Answer without context	$< 1 \times 10^{-50}$	53
			Answer with context	$< 1 \times 10^{-50}$	
		2	Question	$< 1 \times 10^{-50}$	119
			Answer without context	2.3×10^{-10}	105
			Answer with context	$< 1 \times 10^{-50}$	
		3	Question	$< 1 \times 10^{-50}$	81
			Answer without context	2.1×10^{-14}	78
			Answer with context	$< 1 \times 10^{-50}$	
	One-tailed permutation test, with vs. without context	1	Answer (with vs. without context)	1.9×10^{-3}	53
		2	Answer (with vs. without context)	7.9×10^{-5}	105
		3	Answer (with vs. without context)	0.029	78
Classification accuracy	One-tailed bootstrap test, performance vs. chance	1	Question	1.7×10^{-10}	52
			Answer without context	5.9×10^{-7}	
			Answer with context	5.9×10^{-10}	
		2	Question	$< 1 \times 10^{-50}$	101
			Answer without context	4.1×10^{-7}	
			Answer with context	9.7×10^{-14}	
		3	Question	4.0×10^{-14}	75
			Answer without context	2.3×10^{-8}	
			Answer with context	2.7×10^{-13}	
	One-tailed exact McNemar's test, with vs. without context	1	Answer (with vs. without context)	0.033	52
		2	Answer (with vs. without context)	1.9×10^{-6}	101
		3	Answer (with vs. without context)	9.2×10^{-4}	75
Cross entropy	One-tailed bootstrap test, performance vs. chance	1	Question	5.6×10^{-16}	52
			Answer without context	3.3×10^{-3}	
			Answer with context	1.3×10^{-5}	
		2	Question	$< 1 \times 10^{-50}$	101
			Answer without context	1.0×10^{-5}	
			Answer with context	$< 1 \times 10^{-50}$	
		3	Question	$< 1 \times 10^{-50}$	75
			Answer without context	3.7×10^{-11}	
			Answer with context	$< 1 \times 10^{-50}$	
	One-tailed Wilcoxon signed-rank test, with vs. without context	1	Answer (with vs. without context)	7.6×10^{-6}	52
		2	Answer (with vs. without context)	2.6×10^{-17}	101
		3	Answer (with vs. without context)	3.1×10^{-11}	75

[1] The reported number of samples were the number of detected events for the decoding accuracy rate metric and the number of actual trials for the other two metrics.

Supplementary Table 3. Answer classification information transfer rates (ITR; given in bits per second) for each participant.

Participant	Context integration	ITR using full task block durations[1]	ITR using answer speech times[1]	ITR using combined question and answer speech times[1]
1	Without context	0.13	0.90	-
	With context	0.21	1.4	0.43
2	Without context	0.045	0.37	-
	With context	0.11	0.94	0.30
3	Without context	0.085	0.77	-
	With context	0.14	1.3	0.38

[1] All speech times were determined from the acoustic transcriptions, and each task block duration was computed as time interval between the transcribed speech onset time for the first question in the block and the transcribed speech offset time for the final answer in that block.

Supplementary Table 4. Context integration effects on answer classification accuracy for each participant.

Participant	No context (%)[1]	Soft context (%)[1]	Hard context (%)[1]	True context (%)[1]
1	42.31 (−13.46)	55.77 (+0.00)	51.92 (−3.85)	67.31 (+11.54)
2	27.18 (−18.45)	45.63 (+0.00)	44.66 (−0.97)	45.63 (+0.00)
3	38.96 (−15.58)	54.55 (+0.00)	48.05 (−6.49)	59.74 (+5.19)

[1] Each entry specifies the classification accuracy on the answer classification test trials followed by the difference between this accuracy and the corresponding accuracy with soft contexts in parentheses.

Supplementary Table 5. The description and optimization search space for each hyperparameter.

Optimization	Hyperparameter description	Search space type	Value range or choices
Speech Detection	Electrode relevance p-value threshold	Logarithmically uniform	$\left[10^{-50}, 10^{-3}\right]$
	Duration before t to include in the spatiotemporal neural feature vector y_t (in ms)	Uniform	$[1, 300]$
	Duration after t to include in the spatiotemporal neural feature vector y_t (in ms)	Uniform	$[1, 300]$
	Minimum amount of variance the principal components should explain when fitting the PCA model	Uniform	$[0.01, 0.99]$
	Question perception averaging window size (in samples)	Uniform (integer)	$[80, 160]$
	Question perception probability threshold	Uniform	$[0.4, 0.9]$
	Question perception time threshold (in samples)	Uniform (integer)	$[5, 60]$
	Question perception onset index shift (in samples)	Uniform (integer)	$[-100, 100]$
	Question perception offset index shift (in samples)	Uniform (integer)	$[-100, 300]$
	Answer production averaging window size (in samples)	Uniform (integer)	$[20, 80]$
	Answer production probability threshold	Uniform	$[0.4, 0.9]$
	Answer production time threshold (in samples)	Uniform (integer)	$[2, 10]$
	Answer production onset index shift (in samples)	Uniform (integer)	$[-100, 0]$
	Answer production offset index shift (in samples)	Uniform (integer)	$[-100, 50]$
Utterance classification (for questions and answers)	Electrode relevance p-value threshold	Logarithmically uniform	$\left[10^{-50}, 10^{-3}\right]$
	Set of hidden states for the HMMs (S)	Choice	Phones or phonemes[1]
	HMM self-transition probability (p_{self})	Uniform	$[0.1, 0.9]$
	Shift relative to t specifying the first data point in the spatiotemporal neural feature vector y_t (in ms)	Uniform	$[-200, 200]$
	Duration of each spatiotemporal neural feature vector (in ms)	Uniform	$[10, 400]$
	Minimum amount of variance the principal components should explain when fitting the PCA model	Uniform	$[0.01, 0.99]$
	Number of samples of each phone to include when training the phone likelihood models[2]	Uniform (integer)	$[50, 3000]$
	HMM emission probability scaling factor (w_e)	Uniform	$[0.1, 5.0]$
	Log likelihood smoothing factor (ω)	Uniform	$[0.0001, 1.0]$
Context integration	Context prior scaling factor (m)	Logarithmically uniform	$[0.1, 10]$

[1] Phoneme labels were simply the phone labels without stress markings. Across all test blocks and participants, phoneme labels were only deemed optimal for one question classifier and one answer classifier.
[2] This restriction on the number of available samples for each phone was primarily used to allow the optimizer to limit the number of silence tokens included during training (there were much more silence data points than data points for any other phone).

Supplementary notes

Supplementary Note 1. Information transfer rate analysis.

The information transfer rate (ITR) metric quantifies the amount of information that a system communicates per unit time and is commonly used to evaluate brain-computer interfaces[2]. Based on ITR formulations described in existing literature[2,3], we used the following formula to compute ITRs in this work:

$$\text{ITR} = \frac{1}{T}\left[\log_2 N + P\log_2 P + (1-P)\log_2\left(\frac{1-P}{N-1}\right)\right], \tag{1}$$

where N is the number of answer responses in each test trial (which was always equal to 24), P is the prediction accuracy (which was the answer classification accuracy across all of the test blocks for a participant, either with or without context integration), and T is the average time duration for each trial. This formula makes the following assumptions: (1) on average, all possible answer responses have the same prior probability of being the correct answer choice in any trial; (2) the classification accuracy values used for P is stable over time, which should be a valid assumption here given the number of test trials collected with each participant; and (3) on average, each incorrect answer response has an equal probability of being predicted. We used this formula to compute ITRs from the answer classification results using three different methods of measuring trial duration (Supplementary Table 3). We used the transcribed speech onset and offset times during all trial duration calculations.

First, we computed ITRs using full test durations for each participant. In this approach, the value used for T was computed by combining the full test block durations (from the onset of the first question to the offset of the final answer) across all test blocks and then dividing by the total number of test trials. This approach provides a conservative estimate of the ITR by assuming that the entire task duration was necessary for utterance predictions, which results in an increased T and a decreased ITR.

Next, we computed ITRs using the mean utterance duration across the produced answer responses during testing with each participant. In this approach, the value used for T was computed by combining the duration of each answer utterance during testing and dividing by the total number of test trials. This approach provides a liberal estimate of the ITR by assuming that only the time spent by the participant physically producing the utterances were relevant to the ITR calculation, resulting in the highest ITR values observed in this work.

Finally, we computed ITRs using the mean question and answer duration across the test trials for each participant. In this approach, the value used for T was computed by combining the duration of each question utterance and each answer utterance during testing and dividing by the total number of test trials. This approach provides a more moderate estimate of the ITR than the estimate described in the previous paragraph by also including the time required for the participant to listen to the questions. Because the questions were only relevant to answer classification when integrating context, this approach was only used for the answer with context classifications.

In general, the observed decoding accuracy rates are approaching the range that would be useful to patients relying on this technology to communicate. Importantly, these rates reflect

decoding of produced utterances that participants chose to say voluntarily (as opposed to specific cued utterances that were read or repeated on each trial). Although our information transfer rates (ITRs) were not as high as those observed in previous ECoG-based speech classification efforts[3] and intracortical communication prosthetic applications[4], our speech targets consisted of a small set of words or phrases as opposed to letter and syllable targets used in those works. A common criticism of the ITR metric is that it does not consider how easy or natural the behavioral paradigm is[5,6], which currently presents an inherent tradeoff in decoding systems. Unnatural interfaces could lead to increased rates of participant fatigue compared to natural ones, and ITR also does not take into account the time it takes for participants to learn to use the system. Here, we placed a greater emphasis on task naturalness than on maximization of ITR. However, generalizability and accuracy of this more natural decoding approach can be improved in future applications.

Supplementary Note 2. Context integration effects.

We assessed how manipulations to the context integration approach affected answer classification performance. In Section 2.2, we showed that context integration improves decoder performance for each participant. This context integration approach involves using soft context priors, which refers to the fact that soft classification is performed for each question utterance to obtain a probability distribution over the possible questions, which are then used to compute the answer priors. An alternative approach is to use hard context priors, which force the decoded answer in any trial to be the most likely answer utterance within the same question/answer set as the predicted question. To assess the efficacy of using hard priors during context integration, we repeated the context integration step using hard priors for each participant and measured the resulting classification accuracies. We found that answer classification accuracy was always lower when using hard priors instead of soft priors, although this effect was not significant in any participant (participant 1: $p = 0.25$, participant 2: $p = 0.50$, participant 3: $p = 0.063$, one-tailed exact McNemar's test; Supplementary Table 4). These findings suggest that the decoding system would not benefit from greater constraints on which answer utterances are allowed in each trial based on the predicted questions.

We also evaluated how using true priors, determined from the actual presented questions, affected performance. In this approach, the decoded answer is the most likely answer utterance within the same question/answer set as the actual question. To obtain an upper bound on the performance of the context integration models, we repeated the context integration step using true priors for each participant. We found that answer classification accuracy was significantly higher for participant 1, identical for participant 2, and slightly higher (but not significant) for participant 3 when using true priors instead of soft priors (participant 1: $p = 0.016$, participant 2: $p = 1.0$, participant 3: $p = 0.063$, one-tailed exact McNemar's test; Supplementary Table 4). This finding is supported by the relatively high question classification accuracy for participant 2 compared to the other participants, suggesting that the context integration model was performing at its upper bound only for participant 2 and not for the other participants.

Supplementary Note 3. Effect of spatial resolution on decoding performance.

We assessed the impact that the high spatial resolution of the ECoG arrays used with our participants had on performance. To simulate a low-resolution ECoG array, we sub-divided the electrodes for participant 1 into four distinct sets: one set containing the electrodes spatially located in the odd-numbered rows and odd-numbered columns of the ECoG grid, another set containing the electrodes located in the odd-numbered rows and even-numbered columns, and two more sets determined similarly except with even-numbered rows (refer to Fig. 2 for the electrode locations for this participant). For each of these four sets, we evaluated the performance of the system while restricting models to only have access to the electrodes in the current set during training and testing (hyperparameter values from the high resolution models were used here). We found that performance was significantly worse for the low-resolution models compared to the high-resolution ones for each prediction type and performance metric ($p < 0.05$, one-tailed one-sample t-test; Supplementary Figure 4) except for the answer detection score ($p = 0.12$, one-tailed one-sample t-test). These findings emphasize the importance of high spatial resolution when using cortical features to decode speech.

The high spatial resolution of the ECoG arrays used in this work played a major role in our ability to reliably decode speech from cortical activity. Although our findings suggest that low-resolution neural signals could be used to successfully detect speech production events, future neural-based speech decoding efforts should prioritize the use of signal acquisition paradigms with high spatial resolutions to maximize performance.

Supplementary Note 4. Likelihood normalization and the emission probability scaling factor w_e.

In theory, the HMMs used in the utterance classification models require the likelihood values $p(y_t|q_t)$. In practice, however, we obtained phone posteriors $p(q_t|y_t)$ from the LDA models and used these in place of the likelihoods. Because we used flat (uniform) priors over the phone classes in these models, these posteriors were simply equal to the likelihoods after being scaled by an unknown normalization constant. This can be shown via Bayes' rule:

$$p(q_t|y_t) = \frac{p(y_t|q_t)\,p(q_t)}{p(y_t)} = Zp(y_t|q_t)\,, \tag{2}$$

where $p(q_t)$ is a constant because flat priors were used and Z is the unknown constant caused by the presence of the $p(y_t)$ term and the $p(q_t)$ constant.

This discrepancy is addressed by the emission probability scaling factor w_e. By including this hyperparameter, the contribution of the emission probabilities during each iteration of Viterbi decoding (in Eq. 1 in the main text) becomes $w_e Zp(y_t|q_t)$. Because the value of w_e is set through hyperparameter optimization, the impact that this constant Z has on decoding is mitigated. This assumes that the optimizer is capable of finding a satisfactory value of this hyperparameter within its pre-defined range of possible values, which we have observed in practice.

Supplementary Note 5. Mathematical formulation of the context integration model.

During testing, the utterance classification models receive (from the speech detection model) time windows of high gamma features associated with detected question (γ_Q) and detected answer (γ_A) events. A primary goal of these classifiers and the context integration model is to predict the most likely answer utterance $\hat{u}_{a+}$ given the neural features γ_Q and γ_A. This goal can be expressed as:

$$\hat{u}_{a+} = \underset{u_a \in U_A}{\operatorname{argmax}}\, p\left(u_a \mid \gamma_Q, \gamma_A\right), \tag{3}$$

where u_a is one of the answer utterances and U_A is the set of all answer utterances.

This conditional probability $p\left(u_a \mid \gamma_Q, \gamma_A\right)$ represents the posterior probability of u_a given the question-related and answer-related neural features. We can refactor this posterior probability using the following steps (a description of each step is provided after the equations):

$$\begin{aligned}
p\left(u_a \mid \gamma_Q, \gamma_A\right) &= \sum_{u_q \in U_Q} p\left(u_a, u_q \mid \gamma_A, \gamma_Q\right) && (4)\\
&= \sum_{u_q \in U_Q} p\left(u_a \mid u_q, \gamma_A, \gamma_Q\right) p\left(u_q \mid \gamma_A, \gamma_Q\right) && (5)\\
&= \sum_{u_q \in U_Q} p\left(u_a \mid u_q, \gamma_A\right) p\left(u_q \mid \gamma_A, \gamma_Q\right) && (6)\\
&= \sum_{u_q \in U_Q} \frac{p\left(\gamma_A \mid u_a, u_q\right) p\left(u_a \mid u_q\right)}{p\left(\gamma_A \mid u_q\right)} p\left(u_q \mid \gamma_Q, \gamma_A\right) && (7)\\
&= \sum_{u_q \in U_Q} \frac{p\left(\gamma_A \mid u_a\right) p\left(u_a \mid u_q\right)}{p\left(\gamma_A \mid u_q\right)} p\left(u_q \mid \gamma_Q, \gamma_A\right) && (8)\\
&= \sum_{u_q \in U_Q} \frac{p\left(\gamma_A \mid u_a\right) p\left(u_a \mid u_q\right)}{p\left(\gamma_A \mid u_q\right)} \frac{p\left(\gamma_A, \gamma_Q \mid u_q\right) p\left(u_q\right)}{p\left(\gamma_A, \gamma_Q\right)} && (9)\\
&= \sum_{u_q \in U_Q} \frac{p\left(\gamma_A \mid u_a\right) p\left(u_a \mid u_q\right)}{p\left(\gamma_A \mid u_q\right)} \frac{p\left(\gamma_A \mid u_q\right) p\left(\gamma_Q \mid u_q\right) p\left(u_q\right)}{p\left(\gamma_A, \gamma_Q\right)} && (10)\\
&= \sum_{u_q \in U_Q} p\left(\gamma_A \mid u_a\right) p\left(u_a \mid u_q\right) \frac{p\left(\gamma_Q \mid u_q\right) p\left(u_q\right)}{p\left(\gamma_A, \gamma_Q\right)} && (11)\\
&= \frac{1}{p\left(\gamma_A, \gamma_Q\right)} \sum_{u_q \in U_Q} p\left(\gamma_A \mid u_a\right) p\left(u_a \mid u_q\right) p\left(\gamma_Q \mid u_q\right) p\left(u_q\right) && (12)\\
&= \frac{1}{p\left(\gamma_A, \gamma_Q\right) \left|U_Q\right|} \sum_{u_q \in U_Q} p\left(\gamma_A \mid u_a\right) p\left(u_a \mid u_q\right) p\left(\gamma_Q \mid u_q\right) && (13)\\
&= \frac{p\left(\gamma_A \mid u_a\right)}{p\left(\gamma_A, \gamma_Q\right) \left|U_Q\right|} \sum_{u_q \in U_Q} p\left(u_a \mid u_q\right) p\left(\gamma_Q \mid u_q\right) && (14)\\
&\propto p\left(\gamma_A \mid u_a\right) \sum_{u_q \in U_Q} p\left(u_a \mid u_q\right) p\left(\gamma_Q \mid u_q\right) && (15)
\end{aligned}$$

Each step in the above formulation is described below:

- 4: The posterior probability can be expressed as the sum of the joint probability of the answer utterance u_a and question utterance u_q for each question utterance in the set of all question utterances U_Q (while still conditioned on the neural responses).
- 5: The probability can be refactored using the chain rule of probability.
- 6: u_a is independent of γ_Q given u_q.
- 7: The first probability term is refactored using Bayes' theorem.
- 8: γ_A is independent of u_q given u_a.
- 9: The second probability term is refactored using Bayes' theorem.
- 10: One of the terms is refactored into two terms using the fact that γ_A and γ_Q are conditionally independent given u_q.
- 11: A term in the numerator of one fraction cancels the identical term in the denominator of the other fraction.
- 12: The term in the denominator of the remaining fraction can be moved outside of the sum because it does not depend on u_q.
- 13: Because we assume a uniform prior over the question utterances, the $p(u_q)$ term is a constant value equal to 1 divided by the total number of question utterances and can be moved outside of the sum because it does not depend on u_q.
- 14: The first term in the sum is moved outside of the sum since it does not depend on u_q.
- 15: The denominator of the fraction outside of the sum does not depend on u_a, so the posterior probability can be expressed as being proportional to the remaining terms.

The terms in Supplementary Equation 15 are defined below:

- $p(\gamma_A \mid u_a)$ represents the answer likelihoods obtained from the answer classifier.
- $p(\gamma_Q \mid u_q)$ represents the question likelihoods obtained from the question classifier.
- $p(u_a \mid u_q)$ represents the pre-defined context priors.
- $\sum_{u_q \in U_Q} p(u_a \mid u_q)\, p(\gamma_Q \mid u_q)$ represents the answer priors.

In practice, we performed the calculations using log probabilities, and we used a context prior scaling factor m to control the weight of the answer priors relative to the answer likelihoods when computing the answer posteriors. With these modifications, the following formulas can be used to define the unnormalized answer log posterior probabilities:

$$p(u_a \mid \gamma_Q, \gamma_A) \propto p(\gamma_A \mid u_a) \left[\sum_{u_q \in U_Q} p(u_a \mid u_q)\, p(\gamma_Q \mid u_q) \right]^m, \tag{16}$$

$$\phi_{u_a} := \log p\left(u_a \mid \gamma_A, \gamma_Q\right) \tag{17}$$

$$= \log p\left(\gamma_A \mid u_a\right) + m \log\left\{\sum_{u_q \in U_Q} \exp\left[\log p\left(u_a \mid u_q\right) + \log p\left(\gamma_Q \mid u_q\right)\right]\right\} + \kappa \tag{18}$$

$$= \ell^*_{u_a} + m \log\left\{\sum_{u_q \in U_Q} \exp\left[\log p\left(u_a \mid u_q\right) + \ell^*_{u_q}\right]\right\} + \kappa, \tag{19}$$

Each of these additional formula are described below:

- 16: In practice, the formula representing the answer posteriors includes the context prior scaling factor m.
- 17: ϕ_{u_a} is defined as the unnormalized log posterior probability of answer utterance u_a given the neural data γ_Q and γ_A.
- 18: When re-factoring a proportionality equation to an equality using log, a constant scalar value κ is introduced.
- 19: Using notation introduced in the main text (in Eq. 2), we use $\ell^*_{u_a}$ to denote the log likelihood of utterance u_a obtained from the answer classifier and $\ell^*_{u_q}$ to denote the log likelihood of utterance u_q obtained from the question classifier.

In practice, we do not compute the value of κ. We can define a variable to represent the unnormalized log posterior values without κ:

$$\phi'_{u_a} = \phi_{u_a} - \kappa = \ell^*_{u_a} + m \log\left\{\sum_{u_q \in U_Q} \exp\left[\log p\left(u_a \mid u_q\right) + \ell^*_{u_q}\right]\right\} \tag{20}$$

We can then predict the most likely answer utterance $\hat{u}_{a+}$ directly from these ϕ'_{u_a} values:

$$\hat{u}_{a+} = \underset{u_a \in U_A}{\operatorname{argmax}}\, \phi'_{u_a}. \tag{21}$$

Here, κ does not need to be included because it will not affect which answer utterance was most likely.

Although we did not need to normalize the answer log posteriors to predict the most likely answer utterance, we still require normalized log posteriors (normalized to sum to 1 across all answer utterances) when calculating the cross entropy of the answer with context

predictions. We compute normalized answer log posteriors using the following formulation:

$$\phi^*_{u_a} := \phi_{u_a} - \log\left[\sum_{j\in U_A} \exp\left(\phi_j\right)\right] \tag{22}$$

$$= \phi'_{u_a} + \kappa - \log\left[\sum_{j\in U_A} \exp\left(\phi'_j + \kappa\right)\right] \tag{23}$$

$$= \phi'_{u_a} + \kappa - \log\left[\exp\left(\kappa\right)\sum_{j\in U_A} \exp\left(\phi'_j\right)\right] \tag{24}$$

$$= \phi'_{u_a} + \kappa - \log\left[\exp\left(\kappa\right)\right] - \log\left[\sum_{j\in U_A} \exp\left(\phi'_j\right)\right] \tag{25}$$

$$= \phi'_{u_a} - \log\left[\sum_{j\in U_A} \exp\left(\phi'_j\right)\right] \tag{26}$$

Each of these steps is described below:

- 22: $\phi^*_{u_a}$ is defined as the normalized log posterior probability of answer utterance u_a given the neural data γ_Q and γ_A (the term on the right represents the LogSumExp function used to normalize log probabilities).
- 23: We can replace the ϕ_{u_a} terms using our definition of ϕ'_{u_a}.
- 24: Because of the associativity of multiplication, we can express $\exp\left(\phi'_j + \kappa\right)$ as the product of $\exp\left(\kappa\right)$ and $\exp\left(\phi_j\right)$ and then move $\exp\left(\kappa\right)$ out of the sum because it does not depend on j.
- 25: From the logarithmic identity for the logarithm of a product, we can separate the terms in the log function into the sum of the logarithms of the individual terms.
- 26: The $-\log\left[\exp\left(\kappa\right)\right]$ term simplifies to $-\kappa$, which cancels out the κ term.

Supplementary methods

Supplementary Method 1. Utterance decoding sensitivity analysis procedure.

To assess how the amount of available training data affected classification performance, we evaluated classifiers that were trained on varying amounts of data (Fig. 3a). For each participant and utterance type, we first randomly selected $n = 1$ samples of each utterance from the available training data. For each utterance selected this way, we obtained the neural feature vectors and phone labels that occurred between 150 ms before the speech onset and 150 ms after the speech offset. The onset and offset times were determined from the phonetic transcriptions, and the time points before and after each utterance were included so that the classifiers would have sufficient samples of the silence phone /sp/. Utterance classification models were trained using these features and labels associated with the selected utterances (separate models were trained for each test block using the optimized hyperparameter values for that block). We then evaluated the classifiers (and the context integration models) across all of the test blocks using the classification accuracy and cross entropy metrics. We repeated this process 15 times, each time drawing a new random selection of n samples of each utterance to use during training. Afterwards, we then performed all of these steps for every integer value of n in the closed interval $[2, N_{\text{max}}]$, where N_{max} denotes the total number of samples of each utterance available across the training blocks for the participant ($N_{\text{max}} = 10, 30, 20$ for participants 1–3). All random selections of the utterances to use during training were sampled without replacement. We plotted the mean and standard error of the mean of the classification accuracy and cross entropy values across the 15 repeats for each value of n to visualize how classification was affected by the amount of training data.

Similarly, to assess how the amount of available training data affected detection performance, we evaluated speech detectors that were trained on varying amounts of data (Supplementary Figure 6). For each participant, we divided all of the available training data into three subsets containing only the data points occurring during either perception, production, or silence. We then randomly selected 1% of the data points from each of these three subsets (rounding down) and used the random selection to fit detection models for each test block (using the corresponding optimized hyperparameters for each block). Question and answer detection scores were computed for each test block using these models. We repeated this process 10 times, each time drawing a new random 1% selection to use during training. Afterwards, we then performed all of these steps for each of the following percents: 5%, 10%, 25%, 50%, 75%, and 100%. All random data point selections were sampled without replacement. We plotted the mean and standard error of the mean of the detection scores across the 10 repeats for each percent to visualize how detection was affected by the amount of training data.

To better understand the effect that hyperparameter selection had on classifier performance, we evaluated classifiers with many different hyperparameter configurations (Fig. 3b). For each participant, we arbitrarily selected one of the test blocks for that participant and obtained each of the 250 hyperparameter sets that were evaluated (on a separate validation set) during optimization of the question and answer classifiers for that test block. For each of these hyperparameter sets, we trained the utterance classifiers with the configuration using all of the training data available for that participant, and we then evaluated the classifier performance

on the chosen test block using the classification accuracy and cross entropy metrics. We plotted the resulting accuracies and cross entropies for each of these hyperparameter sets (including the configuration that was deemed optimal).

Supplementary Method 2. Hyperparameter optimization procedure.

For each participant, we first performed optimization for the speech detection models. During each optimization epoch, the speech event probability model was trained using all of the available training data and tested on each block in the validation set. As described in Section 4.8.1, we used a custom speech detection score to evaluate the performance of the speech detector (a higher speech detection score signified better performance). The loss function used during speech detection optimization was defined as:

$$\mathcal{L}_{\text{detection}} := \sum_{\beta \in B, \psi \in \{\text{question,answer}\}} \left(1 - s^2_{\text{detection},\beta,\psi}\right), \tag{27}$$

where $\mathcal{L}_{\text{detection}}$ is the detection loss, β signifies one of the blocks in the validation set B, ψ signifies one of the utterance types (either question or answer), and $s_{\text{detection},\beta,\psi}$ is the speech detection score associated with validation block β and utterance type ψ. Thus, the optimal hyperparameters for the speech detection model associated with each test block were the hyperparameters that best detected the question and answer events in the validation blocks.

Next, we performed optimization for the utterance classification models. Separate optimizations were performed for the question and answer classifiers. During each optimization epoch, the phone likelihood models were trained using all of the available training data. Afterwards, the utterance classifiers predicted the utterance labels of the speech events that were detected by the optimized speech detection models in each validation block. We used cross entropy on the validation set as the loss function during optimization. Although the true speech event times were used during cross entropy calculations in other analyses, we chose to optimize the classifiers using the detected times to increase the robustness of the classifiers to imperfect speech event detection. To compute cross entropy on the decoded utterances from the detected events, we had to first convert the decoded sequence to classification trials. We performed this conversion by iterating through the actual utterances in chronological order and pairing each actual utterance label with the detected utterance label that had the closest detected speech offset time to the actual speech offset time (pairing a detected utterance label with more than one actual label was prevented). The optimal hyperparameters for each utterance classification model associated with each test block were the hyperparameters that resulted in the lowest cross entropy on the detected speech events in the validation blocks.

Finally, we optimized the context integration models. The only goal of this optimization process was to choose a value for the context prior scaling factor m. For each test block, we decoded utterance sequences in the validation blocks using the optimized speech detection and utterance classification models. During each optimization epoch, the answer with context predictions were computed using the decoded question and answer (without context) sequences and the current value of the hyperparameter m. Similar to the utterance classifier optimization, we used cross entropy on the validation set as the loss function during optimization. The decoded answer with context sequences were converted to classification trials so that the cross entropy could be computed. The optimal value of m for the context integration model associated with each test block was the value that resulted in the lowest cross entropy of the answer with context predictions using the detected speech events in the validation blocks.

Supplementary Method 3. Utterance classifier phonetic characterization procedure.

To visualize the process by which the classifiers used Viterbi decoding to update the predicted likelihood of each utterance as it received additional neural data, we examined how the utterance likelihoods changed over time during a correctly predicted answer utterance for one of the participants (Fig. 4a). Using the time window of neural activity associated with that utterance and the trained phone likelihood model for the test block containing the selected trial, we performed Viterbi decoding on the HMM for each answer utterance. At each time index t during the Viterbi decoding with each HMM, we stored the log likelihood of the most likely Viterbi path through the HMM at that time index given the neural feature vectors $\{y_0, y_1, \ldots, y_t\}$ (where y_0 is the first feature vector in the time window). Afterwards, the path likelihoods at each time point were smoothed (using ω, the smoothing hyperparameter from the classifier) and normalized (to sum to 1) across all utterances. The resulting values were plotted as the probability of each utterance at each time point during Viterbi decoding.

We also used these path probabilities to measure the amount of time points required before each classifier finalized its prediction of which utterance was most likely during each trial (Fig. 4b). Across all test blocks and participants, we computed the Viterbi path probabilities at each time point during classification of each answer trial. For each trial, we used these path probabilities to find the earliest time index at which the predicted utterance (the utterance with the highest path probability at the final time index) became and remained more likely than all of the other utterances (denoted $t_{\text{finalization}}$). We computed the decision finalization time for a trial using the following formula:

$$\tau = \frac{t_{\text{finalization}} - t_{\text{onset}}}{t_{\text{offset}} - t_{\text{onset}}}, \tag{28}$$

where τ is the decision finalization time and t_{onset} and t_{offset} are the speech onset and offset time indices of the utterance, respectively (obtained from the phonetic transcriptions). To assess how well the decision finalization times could be explained by the phonetic content and pronunciation of the stimuli, we also computed these values using phone likelihoods constructed directly from the phonetic transcriptions (without using neural data to infer the phone likelihoods). For each trial, we provided as input to the Viterbi decoder for each HMM a time series of phone likelihoods in which, at each time point, the probability of the phone that was actually occurring (according to the phonetic transcriptions) was equal to 0.9 and the remaining 0.1 probability mass was divided evenly among the other phones. During Viterbi decoding, all of the non-zero phone transition probabilities $p\left(q_{t+1} | q_t\right)$ for each HMM were set equal to 0.5. To compare the finalization times between the neural-based and transcription-based analyses, we only considered trials in which the neural-based classifier correctly predicted the utterance identity (we did not find a significant difference in finalization times between the correct and incorrect trials, $p = 0.37$, two-tailed Welch's t-test). In the decision finalization time plot, trials in which the finalization time was negative for the neural-based model were excluded (5 trials were excluded from and 89 trials were included in the figure).

To assess how well the answer phone likelihood models were able to discriminate between the phonetic classes, we computed phone confusions across all of the test blocks and

participants (Fig. 4c). For each test block, we used the phone likelihood model within the associated answer classification model to predict the phone label at each time point. We compared the actual and predicted phone labels at each time point across all test blocks to compute the plotted phone confusion matrix. We excluded one test block for participant 2 from this analysis because the answer classification model associated with that test block used phonemic labels (labels without stress markers) instead of the phonetic labels used in all of the other test blocks (see Supplementary Table 5). Using these actual and predicted phone labels, we also assessed whether or not the phone confusions were at least partially organized by place of articulation. We collapsed the phone labels into category labels using the place of articulation categorization depicted in Fig. 4c (9 phonetic categories). Time points that did not occur during speech production were excluded, although silence could still appear in the predicted labels for misclassified time points (these were assigned a special category label different from the other 9). We then computed the mutual information between the actual and predicted category label sequences[7]. This approach was also used to compute the mutual information values with randomized phonetic categorizations during statistical significance testing.

Supplementary Method 4. Statistical testing.

When comparing the decoding accuracy rates to chance (Fig. 2a, Supplementary Figure 3a), we used a one-tailed bootstrap test. First, for each participant and prediction type (questions, answers without context, and answers with context), we concatenated the actual and predicted utterances across all of the test blocks to obtain overall actual and predicted utterance sequences. We then created a sequence with length equal to the number of utterance tokens in the overall actual sequence and with elements randomly sampled from the set of possible utterance labels for the current prediction type (sampling was done with replacement and with a uniform probability distribution across the possible labels). Next, we computed the decoding accuracy rate by comparing this random sequence with the actual sequence. We performed this process of creating a random sequence and computing its accuracy rate one million times. Afterwards, we created a normal distribution parameterized by the mean and standard deviation of the accuracy rates observed during this process. We determined the value of the cumulative distribution function (CDF) of this distribution at the value equal to the decoding accuracy rate associated with the current test block and prediction type. The one-tailed p-value for the null hypothesis that the decoded accuracy rate was not above chance was equal to 1 minus this CDF value. Our method of measuring chance performance was arguably an overestimate of the true chance performance because it uses the same sequence length as the actual utterance sequence within a test block (this is equivalent to assuming that the speech detector always detected the correct number of events).

When comparing the answer with context and answer without context decoding accuracy rates (Fig. 2a, Supplementary Figure 3a), we used a one-tailed permutation test. First, for each participant, we obtained the overall actual answer sequence and the overall predicted answer without context and answer with context sequences by concatenating across test blocks (as previously described). The two predicted answer sequences were always the same length (each of these types of predictions were made every time an answer event was detected during testing). We then created a mixture predicted sequence of the same length as these two sequences in which the value at any index i was randomly selected as either the utterance label at index i in the without context decoded sequence or the label at index i in the with context decoded sequence (with an equal probability of choosing from either). Next, we computed the decoding accuracy rate for this mixture sequence. We performed this process of randomly creating a mixture sequence and computing its accuracy rate one million times. Afterwards, we created a normal distribution parameterized by the mean and standard deviation of the accuracy rates observed during this process. We determined the value of the CDF of this distribution at the value equal to the decoding accuracy rate associated with the answer with context predictions in the current test block. The one-tailed p-value for the null hypothesis that the accuracy rate of the answer predictions with context was not above the predictions without context was equal to 1 minus this CDF value.

When comparing the classification accuracies to chance (Fig. 2b, Supplementary Figure 3b), we used a one-tailed bootstrap test. First, for each participant and prediction type, we created a sequence with length equal to the number of trials across all test blocks and with a randomly sampled utterance (from the set of possible utterances for the current prediction type) as each element. We then computed a chance classification accuracy value by comparing these randomly selected utterance labels to the actual utterance labels. We performed this

process of creating a random predicted sequence and computing the classification accuracy one million times. Next, using the classification predictions observed during testing, we computed a correctness indicator array that contained 1 for each classification trial (across all test blocks) in which the actual and predicted labels were equal and 0 for the remaining trials. We then created a sequence with length equal to the number of trials across all test blocks and with elements randomly sampled from this correctness array (sampling was done with replacement). Next, we computed the classification accuracy as the mean of this random resample of the correctness array. We performed this process of randomly resampling the correctness array and computing the classification accuracy one million times. Afterwards, we created a normal distribution parameterized by the mean and standard deviation of the classification accuracies observed during this process (the accuracies computed from the resamples of the correctness array). We determined the value of the CDF of this distribution at the value equal to the mean chance classification accuracy (the mean of the accuracies computed from the randomly sampled utterance sequences). This CDF value was used as the one-tailed p-value for the null hypothesis that the predicted classification accuracies were not higher than chance.

When comparing the answer with context and answer without context classification accuracies (Fig. 2b, Supplementary Figure 3b), we used a one-tailed exact McNemar's test. First, for each participant, we obtained the correctness indicator arrays described earlier for the with context and without context answer predictions. We then used a one-tailed exact McNemar's test to compare these two arrays[8]. McNemar's test is suited for comparing two paired binary sequences. In this application of McNemar's test, the number of trials that were correctly predicted when using context but incorrect without context are compared to the number of trials that were correctly predicted without using context but incorrect with context. The resulting p-value from this test was the probability of the null hypothesis that the classification accuracy was not higher for answer predictions with context than those without context.

When comparing cross entropies to chance (Fig. 2c, Supplementary Figure 3c), we used a one-tailed bootstrap test. First, for each participant and prediction type, we computed the negative predicted log probability values associated with the actual utterance label within each classification trial (across all test blocks). These negative log probability values are referred to as surprisals. Then, we created a new array with length equal to the number of trials across all test blocks. Each element in this array was randomly sampled from the array of surprisal values associated with the predictions (sampling was done with replacement and with a uniform probability distribution across all of the surprisals). We then computed the predicted cross entropy by taking the mean of these randomly sampled surprisals. We performed this process of randomly sampling the surprisals and computing the cross entropy one million times. Afterwards, we created a normal distribution parameterized by the mean and standard deviation of these predicted cross entropy values. We determined the value of the CDF of this distribution at the value equal to the chance cross entropy value, which was computed as the negative log of the reciprocal of the number of possible labels for the current prediction type. The one-tailed p-value for the null hypothesis that the predicted cross entropy was not lower than chance was equal to 1 minus this CDF value (lower cross entropy indicates better performance).

When comparing the answer with context and answer without context cross entropies

(Fig. 2c, Supplementary Figure 3c), we used a one-tailed Wilcoxon signed-rank test. First, for each participant, we obtained the surprisal arrays described earlier for the with context and without context answer predictions. We then used a one-tailed Wilcoxon signed-rank test to compare these paired samples. The resulting p-value from this test was the probability of the null hypothesis that the cross entropy was not lower for answer predictions with context than those without context.

When comparing the low-resolution performance results (computed with sub-sampled electrode sets) to the regular high-resolution performance results for participant 1, we used a one-tailed one-sample t-test. First, for each evaluation metric and prediction type, we computed the performance using models trained and tested with each of the four sub-sampled electrode sets. We then used a one-sample t-test to compare the four low-resolution performance value samples to the high-resolution performance value. The resulting one-tailed p-value from this test was the probability of the null hypothesis that the low-resolution performance was not worse than the high-resolution performance.

When comparing the decision finalization times for the answer classifiers to the speech offset time (Fig. 4b), we used a one-tailed single-sample Wilcoxon signed-rank test. This test was performed on an array created by subtracting each decision finalization time (scaled such that 0 was the speech onset time and 1 was the speech offset time) from the speech offset time (which was 1 due to this scaling). We then performed a one-tailed Wilcoxon signed-rank test to compare the values in this array to zero. The resulting p-value from this test was the probability of the null hypothesis that the decision finalization times for the answer classifiers did not occur before the speech offset.

When comparing the neural-based and transcription-based decision finalization times (Fig. 4b), we used a two-tailed Wilcoxon signed-rank test. This test was performed using the paired decision finalization time samples (a neural-based and transcription-based finalization time was available for each trial used in this test). The resulting p-value from this test was the probability of the null hypothesis that the neural-based and transcription-based finalization times were both sampled from the same underlying distribution.

When comparing the confusions categorized by place of articulation to random categorizations (Fig. 4c), we used a one-tailed bootstrap test. After collapsing the actual and predicted answer phone labels (computed for each time point that occurred during speech production across all participants and test blocks) into the 9 disjoint phonetic categories, we computed the mutual information between the actual and predicted labels[7]. Next, we randomized the phonetic categorization by shuffling which phones appeared in which categories (the total number of phones in each category remained the same) and recomputed the mutual information between the actual and predicted sequences using this new random categorization. We performed this process of computing the mutual information with randomized phonetic categorizations one million times. Afterwards, we created a normal distribution parameterized by the mean and standard deviation of the randomized mutual information values observed during this process. We determined the value of the CDF of this distribution at the value equal to the original mutual information (with the categorization based on place of articulation). The one-tailed p-value for the null hypothesis that the mutual information for the categorization based on place of articulation was not higher than random categorizations was equal to 1 minus this CDF value.

When comparing classification performance using hard or true context priors instead of

soft context priors (Supplementary Note 2), we used one-tailed exact McNemar's tests. For these tests, we used the same approach that was used to compare the answer with context and answer without context classification accuracies (described earlier in this section). When evaluating the hard priors, the classifications made with hard priors replaced the answer without context predictions, and then the remainder of the statistical testing was carried out as previously described. When evaluating the true priors, the classifications made with true priors replaced the answer with context predictions and the classifications made with soft priors replaced the answer without context predictions, and then the remainder of the statistical testing was carried out as previously described.

Supplementary references

[1] Moses, D. A., Leonard, M. K. & Chang, E. F. Real-time classification of auditory sentences using evoked cortical activity in humans. *J. Neural Eng.* **15** (2018).

[2] Wolpaw, J. R., Birbaumer, N., McFarland, D. J., Pfurtscheller, G. & Vaughan, T. M. Brain-computer interfaces for communication and control. *Clin. Neurophysiol.* **113**, 767–91 (2002).

[3] Mugler, E. M. et al. Direct classification of all American English phonemes using signals from functional speech motor cortex. *J. Neural Eng.* **11**, 035015 (2014).

[4] Pandarinath, C. et al. High performance communication by people with paralysis using an intracortical brain-computer interface. *Elife* **6**, 1–27 (2017).

[5] Seno, B. D., Matteucci, M. & Mainardi, L. The Utility Metric : A Novel Method to Assess the Overall Performance of Discrete Brain-Computer Interfaces. *IEEE Trans. Neural Syst. Rehabil. Eng.* **18**, 20–28 (2010).

[6] Spüler, M., Rosenstiel, W. & Bogdan, M. Online Adaptation of a c-VEP Brain-Computer Interface(BCI) Based on Error-Related Potentials and Unsupervised Learning. *PLoS One* **7** (2012).

[7] Thomas, C. & Joy, T. *Elements of Information Theory* (Willey-Interscience, 2006), 2nd edn.

[8] McNemar, Q. Note on the sampling error of the difference between correlated proportions or percentages. *Psychometrika* **12**, 153–157 (1947).
