# Supplementary material for: Real-time decoding of question-and-answer speech dialogue using human cortical activity
Source: Nat Commun. 2019 Jul 30;10:3096. doi: 10.1038/s41467-019-10994-4 (PMC6667454; doi:10.1038/s41467-019-10994-4)
Supplement: Supplementary file 3 — Reporting Summary [file 41467_2019_10994_MOESM3_ESM.pdf]

nature research

Corresponding author(s): *Double-blind peer review submissions: write DBPR and your manuscript number here instead of author names.*

Last updated by author(s): *YYYY-MM-DD*

Reporting Summary

Nature Research wishes to improve the reproducibility of the work that we publish. This form provides structure for consistency and transparency in reporting. For further information on Nature Research policies, see Authors & Referees and the Editorial Policy Checklist.

Statistics

For all statistical analyses, confirm that the following items are present in the figure legend, table legend, main text, or Methods section.

n/a	Confirmed	
☐	☒	The exact sample size (*n*) for each experimental group/condition, given as a discrete number and unit of measurement
☐	☒	A statement on whether measurements were taken from distinct samples or whether the same sample was measured repeatedly
☐	☒	The statistical test(s) used AND whether they are one- or two-sided<br>*Only common tests should be described solely by name; describe more complex techniques in the Methods section.*
☒	☐	A description of all covariates tested
☐	☒	A description of any assumptions or corrections, such as tests of normality and adjustment for multiple comparisons
☐	☒	A full description of the statistical parameters including central tendency (e.g. means) or other basic estimates (e.g. regression coefficient) AND variation (e.g. standard deviation) or associated estimates of uncertainty (e.g. confidence intervals)
☐	☒	For null hypothesis testing, the test statistic (e.g. *F*, *t*, *r*) with confidence intervals, effect sizes, degrees of freedom and *P* value noted<br>*Give P values as exact values whenever suitable.*
☒	☐	For Bayesian analysis, information on the choice of priors and Markov chain Monte Carlo settings
☒	☐	For hierarchical and complex designs, identification of the appropriate level for tests and full reporting of outcomes
☒	☐	Estimates of effect sizes (e.g. Cohen's *d*, Pearson's *r*), indicating how they were calculated

Our web collection on statistics for biologists contains articles on many of the points above.

Software and code

Policy information about availability of computer code

Data collection	TDT OpenEx software
Data analysis	Python 3.6.3, numpy 1.14.2, scipy 1.0.0, scikit-learn 0.19.1, matplotlib 2.2.2, Qt 5, PyQt5, pyqtgraph 0.10.0, HDF 5, h5py 2.7.0, editdistance 0.4, hyperopt 0.1, realtime 0.1.9<br>The realtime package is custom software that can be made available to reviewers upon request.<br>Note that some packages might have been updated as appropriate throughout the timespan of data analysis. The versions given here are the latest versions that were used.

For manuscripts utilizing custom algorithms or software that are central to the research but not yet described in published literature, software must be made available to editors/reviewers. We strongly encourage code deposition in a community repository (e.g. GitHub). See the Nature Research guidelines for submitting code & software for further information.

Data

Policy information about availability of data

All manuscripts must include a data availability statement. This statement should provide the following information, where applicable:

- Accession codes, unique identifiers, or web links for publicly available datasets
- A list of figures that have associated raw data
- A description of any restrictions on data availability

Deidentified copies of the data used in these analyses can be provided upon reasonable request. Please contact the corresponding author via email with any inquiries about the data.

Field-specific reporting

Please select the one below that is the best fit for your research. If you are not sure, read the appropriate sections before making your selection.

☒ Life sciences ☐ Behavioural & social sciences ☐ Ecological, evolutionary & environmental sciences

For a reference copy of the document with all sections, see nature.com/documents/nr-reporting-summary-flat.pdf

Life sciences study design

All studies must disclose on these points even when the disclosure is negative.

Sample size	For our classification performance analyses, the number of samples was typically equal to the number of utterances heard or said by each of the participants. Although the sample sizes typically depended on clinical time constraints, we observed significant trends with these sample sizes.
Data exclusions	Data exclusions were rare, with less than 0.5% of testing trials excluded from analyses due to participant error.
Replication	We observed significant trends for all three of our participants.
Randomization	During the statistical tests based on bootstrapping, we used a million random samples (with replacement) of the trials for each participant.
Blinding	Blinding was not relevant to this study. We did ensure that the data used to train models remained separate from the test data. For each participant, the data was split based on task type into training and testing datasets. Additionally, we used a cross-validated approach when optimizing hyperparameters for the models to maintain separation between training and testing data.

Reporting for specific materials, systems and methods

We require information from authors about some types of materials, experimental systems and methods used in many studies. Here, indicate whether each material, system or method listed is relevant to your study. If you are not sure if a list item applies to your research, read the appropriate section before selecting a response.

Materials & experimental systems

n/a	Involved in the study	
☒	☐	Antibodies
☒	☐	Eukaryotic cell lines
☒	☐	Palaeontology
☒	☐	Animals and other organisms
☐	☒	Human research participants
☒	☐	Clinical data

Methods

n/a	Involved in the study	
☒	☐	ChIP-seq
☒	☐	Flow cytometry
☒	☐	MRI-based neuroimaging

Human research participants

Policy information about studies involving human research participants

Population characteristics	All three participants were patients at the UCSF Medical Center undergoing epilepsy treatment. Electrocorticography arrays were implanted on the cortical surface of each participant for the clinical purpose of localizing seizure foci. All participants were right-handed with left hemisphere language dominance. Two participants were male and one participant was female.
Recruitment	Prior to surgical implantation of the electrocorticography arrays, each patient gave his or her written informed consent to participate in this research.
Ethics oversight	The research protocol was approved by the UCSF Committee on Human Research.

Note that full information on the approval of the study protocol must also be provided in the manuscript.
