# Supplementary material for: Real-time decoding of question-and-answer speech dialogue using human cortical activity
Source: Nat Commun. 2019 Jul 30;10:3096. doi: 10.1038/s41467-019-10994-4 (PMC6667454; doi:10.1038/s41467-019-10994-4)
Supplement: Supplementary file 4 — Description of Additional Supplementary Files [file 41467_2019_10994_MOESM4_ESM.pdf]

Description of Additional Supplementary Files

File Name: Supplementary Movie 1
Description: **Real-time speech decoding.**
This video portrays the real-time decoding task using simulated data from participant 1. An MRI reconstruction of this participant's brain surface is shown with superimposed electrode positions. High gamma magnitudes for the electrodes are plotted throughout the simulation (darker colors signify increased neural activity). The question stimuli (female speaker) and participant responses (male speaker) are included as audio. Ellipses signify speech events detected from neural activity by the speech detector.
